# Perseveration and Shifting in Obsessive-Compulsive Disorder as a Function of Uncertainty, Punishment, and Serotonergic Medication

**DOI:** 10.1016/j.bpsgos.2023.06.004

**Published:** 2023-07-13

**Authors:** Annemieke M. Apergis-Schoute, Febe E. van der Flier, Samantha H.Y. Ip, Jonathan W. Kanen, Matilde M. Vaghi, Naomi A. Fineberg, Barbara J. Sahakian, Rudolf N. Cardinal, Trevor W. Robbins

**Affiliations:** a School of Biological and Behavioural Sciences, Queen Mary University of London, London, United Kingdom; b Behavioural and Clinical Neuroscience Institute, University of Cambridge, Cambridge, United Kingdom; c Centre for Cancer Genetic Epidemiology, Department of Public Health and Primary Care, University of Cambridge, Cambridge, United Kingdom; d British Heart Foundation Cardiovascular Epidemiology Unit, Department of Public Health and Primary Care, University of Cambridge, Cambridge, United Kingdom; e Victor Phillip Dahdaleh Heart and Lung Research Institute, University of Cambridge, Cambridge, United Kingdom; f Department of Psychology, University of Cambridge, Cambridge, United Kingdom; g School of Psychology, University of East Anglia, Norwich, United Kingdom; h Hertfordshire Partnership University NHS Foundation Trust, National Health Service, University of Hertfordshire, Hatfield, United Kingdom; i Department of Psychiatry, University of Cambridge, Cambridge, United Kingdom; j Cambridgeshire and Peterborough NHS Foundation Trust, Cambridge, United Kingdom

**Keywords:** OCD, Perseveration, Punishment, Serotonin, Shifting, Uncertainty

## Abstract

**Background:**

The nature of cognitive flexibility deficits in obsessive-compulsive disorder (OCD), which historically have been tested with probabilistic reversal learning tasks, remains elusive. Here, a novel deterministic reversal task and inclusion of unmedicated patients in the study sample illuminated the role of fixed versus uncertain rules/contingencies and of serotonergic medication. Additionally, our understanding of probabilistic reversal was enhanced through theoretical computational modeling of cognitive flexibility in OCD.

**Methods:**

We recruited 49 patients with OCD, 21 of whom were unmedicated, and 43 healthy control participants matched for age, IQ, and gender. Participants were tested on 2 tasks: a novel visuomotor deterministic reversal learning task with 3 reversals (feedback rewarding/punishing/neutral) measuring accuracy/perseveration and a 2-choice visual probabilistic reversal learning task with uncertain feedback and a single reversal measuring win-stay and lose-shift. Bayesian computational modeling provided measures of learning rate, reinforcement sensitivity, and stimulus stickiness.

**Results:**

Unmedicated patients with OCD were impaired on the deterministic reversal task under punishment only at the first and third reversals compared with both control participants and medicated patients with OCD, who had no deficit. Perseverative errors were correlated with OCD severity. On the probabilistic reversal task, unmedicated patients were only impaired at reversal, whereas medicated patients were impaired at both the learning and reversal stages. Computational modeling showed that the overall change was reduced feedback sensitivity in both OCD groups.

**Conclusions:**

Both perseveration and increased shifting can be observed in OCD, depending on test conditions including the predictability of reinforcement. Perseveration was related to clinical severity and remediated by serotonergic medication.

SEE COMMENTARY ON PAGE 105

The cardinal symptoms of obsessive-compulsive disorder (OCD) are obsessions (unwanted, distressing, recurrent, or persistent intrusive thoughts, images, or urges that the patient cannot control) and compulsions (unwanted repetitive behaviors or mental acts performed according to rigid rules) (1). Therefore, cognitive inflexibility, manifested as perseveration in thought or action, may be an underlying tendency in these OCD symptoms (2, 3, 4). Preclinical work with experimental animals and humans, using reversal rule learning to measure behavioral flexibility, has consistently shown an important role for serotonin (5, 6, 7, 8). Serotonin depletion in the orbitofrontal cortex causes perseveration (9), and such deficits are remediated by selective serotonin reuptake inhibitors (SSRIs) (5), the first-line pharmacological treatment for OCD (10).

Although reversal learning has been associated with orbitofrontal cortex underactivation in OCD, it has proven difficult to demonstrate consistent behavioral deficits in reversal (11). This may be because of the relatively simple, deterministic nature of the basic task: a 2-choice reversal rule-learning task requires an easily detected shift of contingencies, from 100%:0% reinforcement for the 2 options to 0%:100%. Therefore, in the current study, we enhanced the load or difficulty of the deterministic rule-learning task by increasing the number of stimulus-response mappings (fingers on each hand) and added 3 hand-rule reversals in a speeded version of the task. Given evidence in OCD for greater impairments in cognitive performance under conditions of punishment for incorrect responding (12,13), we also compared explicitly punishing feedback with explicit reward for this deterministic rule-learning/reversal task.

Fradkin *et al.* (14) recently postulated that patients with OCD are impaired in their ability to mediate state transitions from one situation to another. Their modeling shows the opposite effects on behavior that this may have in familiar, well-established circumstances compared with uncertain, volatile scenarios for patients with OCD, leading to perseveration in the former and vacillation (increased shifting) in the latter. Therefore, we tested this hypothesis by measuring behavioral performance not only on the novel deterministic rule-learning procedure that we developed (100:0 to 0:100 reinforcement) but also on a classic 2-stimulus probabilistic reversal (80:20 to 20:80 reinforcement) task (15). Hauser *et al.* (16) have hypothesized that people with OCD show a reduced tolerance to uncertainty in probabilistic learning, and Kanen *et al.* (12) used computational modeling of a probabilistic task with multiple reversals to show significant deficits in medicated OCD associated with greater vacillation of decision making or response switching (reduced stickiness) as predicted by Fradkin *et al.* (14). In addition to conventional analyses for both paradigms, we used computational modeling to extract more sensitive measures of performance in the probabilistic reversal task, including stickiness, the tendency to repeat responding to the immediately previous stimulus regardless of feedback (12,17). We examined the effects of SSRIs on both deterministic and probabilistic reversal performance by comparing groups of medicated versus unmedicated patients with OCD.

## Methods and Materials

### Participants

Healthy control participants (*n* = 43), unmedicated patients with OCD (*n* = 21), and medicated patients with OCD (*n* = 28) participated in the study. They were compensated with the chance to earn additional money on the deterministic reversal task based on their performance. The groups were matched for age, gender, verbal intelligence, and handedness. Medicated patients with OCD were referred by psychiatrists in the Hertfordshire Partnership, Cambridgeshire and Peterborough, and South Essex Partnership Foundation Trusts. Unmedicated patients with OCD were recruited via OCD Action and a Cambridge clinical psychologist specializing in OCD (Dr. J. van Niekerk). The majority of unmedicated patients with OCD were medication naïve, and 8 patients had stopped taking medication more than 6 months before taking part in our study. All patients were screened with the Mini-International Neuropsychiatric Interview, and only patients with OCD without an additional Axis I disorder present and a minimum Yale-Brown Obsessive Compulsive Scale (Y-BOCS) score of 12 were included. Demographic and clinical characteristics of both groups are summarized in Table 1. All medicated patients except one were taking an SSRI; one patient’s treatment was also augmented with an antipsychotic (risperidone), and one patient was medicated with clomipramine (the serotonergic tricyclic antidepressant drug) (see Table S5 for individual medication details). All participants gave written informed consent. Post hoc tests confirmed that medicated and unmedicated patients with OCD did not differ on any of the demographic or clinical measures including the Y-BOCS score. Because medicated and unmedicated patients with OCD were matched on the clinical index of OCD severity, it is likely that medicated patients had more severe underlying OCD symptoms because their serotonergic medication was confirmed by the consultant psychiatrist to be effective in reducing OCD symptoms in all cases, and all patients’ condition had stabilized on medication, resulting in a minimum Y-BOCS score of 12. Age at diagnosis was comparable for medicated patients with OCD (mean = 23.23 years, SD = 6.8) and unmedicated patients with OCD (mean = 24.00 years, SD = 7.8) (*p* = .73). Data on illness duration was incomplete and reported verbally by patients. These estimates suggested that illness duration was longer in the medicated OCD group (mean = 14.8 years, SD = 9.6, *n* = 27) than the unmedicated group (mean = 9.85, SD = 7.4, *n* = 20). Age of onset differed less between medicated (mean = 12.5 years, SD = 5.7) and unmedicated (mean = 14.3 years, SD = 9.2) patients.

**Table 1:** Demographics and Mean Scores per Group and Statistical Comparisons

Variable	Control Group, *n* = 43	OCD Medicated Group, *n* = 28	OCD Unmedicated Group, *n* = 21	*F*_89_ Test	*p* Value
Age, Years	37 (11.5)	37 (11.8)	33 (7.3)	1.444	.241
Sex, Female/Male	22/21	14/14	13/8	0.403	.669
NART, Errors	14.69 (6.9)	17.54 (8.0)	16.15 (6.4)	1.311	.275
Education, Years	16.23 (3.2)	15.64 (3.4)	17.33 (2.4)	1.815	.169
Handedness, Right/Left	38/5	24/4	19/2	0.129	.879
Y-BOCS	0.49 (0.6)	22.97 (5.3)	21.19 (5.6)	325.355	.0001^a^
MADRS	0.7 (1.2)	9.11 (5.1)	6.86 (4.88)	46.969	.0001^a^
STAI-State	27.02 (7.8)	40.79 (10.3)	42.86 (10.8)	28.428	.0001^a^
STAI-Trait	33.26 (7.7)	55.82 (9.7)	53.00 (8.5)	72.141	.0001^a^
OCI-R	4.63 (4.3)	34.86 (13.0)	30.71 (12.3)	99.228	.0001^a^
EPI Extraversion	15.88 (3.8)	12.18 (4.5)	12.81 (4.3)	8.083	.001^b^
EPI Neuroticism	7.02 (4.8)	15.04 (4.8)	16.19 (4.3)	37.514	.0001^a^

Values are presented as mean (SD) or *n*. The OCD medicated and unmedicated groups did not differ significantly on any measure.

EPI, Eysenck Personality Inventory; MADRS, Montgomery–Åsberg Depression Rating Scale; NART, National Adult Reading Test; OCI-R, Obsessive Compulsive Inventory–Revised; STAI, State-Trait Anxiety Inventory; Y-BOCS, Yale-Brown Obsessive Compulsive Scale.

a *p* < .0001.

b *p* < .001.

### Questionnaires

Clinical questionnaires were administered verbally; these included the Y-BOCS (18) and Montgomery–Åsberg Depression Rating Scale (19) as well as the National Adult Reading Test (20) to measure verbal intelligence. We used computerized versions of the Spielberger State-Trait Anxiety Inventory (21), the Obsessive Compulsive Inventory–Revised (22), and the Eysenck Personality Inventory (23). Patients with OCD scored significantly higher than control participants on the Y-BOCS, Montgomery–Åsberg Depression Rating Scale, Spielberger State-Trait Anxiety Inventory (state and trait), Obsessive Compulsive Inventory–Revised, and Eysenck Personality Inventory neuroticism and extraversion questionnaires (*p* < .001).

### Apparatus

The stimuli were presented on a 19-inch monitor with a resolution of 1024 × 768 pixels. The deterministic reversal learning experiment was programmed using E-Prime 2.0 software (24). The hand response boxes were specifically designed for this novel deterministic reversal task by the University of Cambridge Biotronix Workshop (Figure S1). The probabilistic reversal task was conducted on a touchscreen computer.

### Deterministic Reversal Task

**Figure 1:**
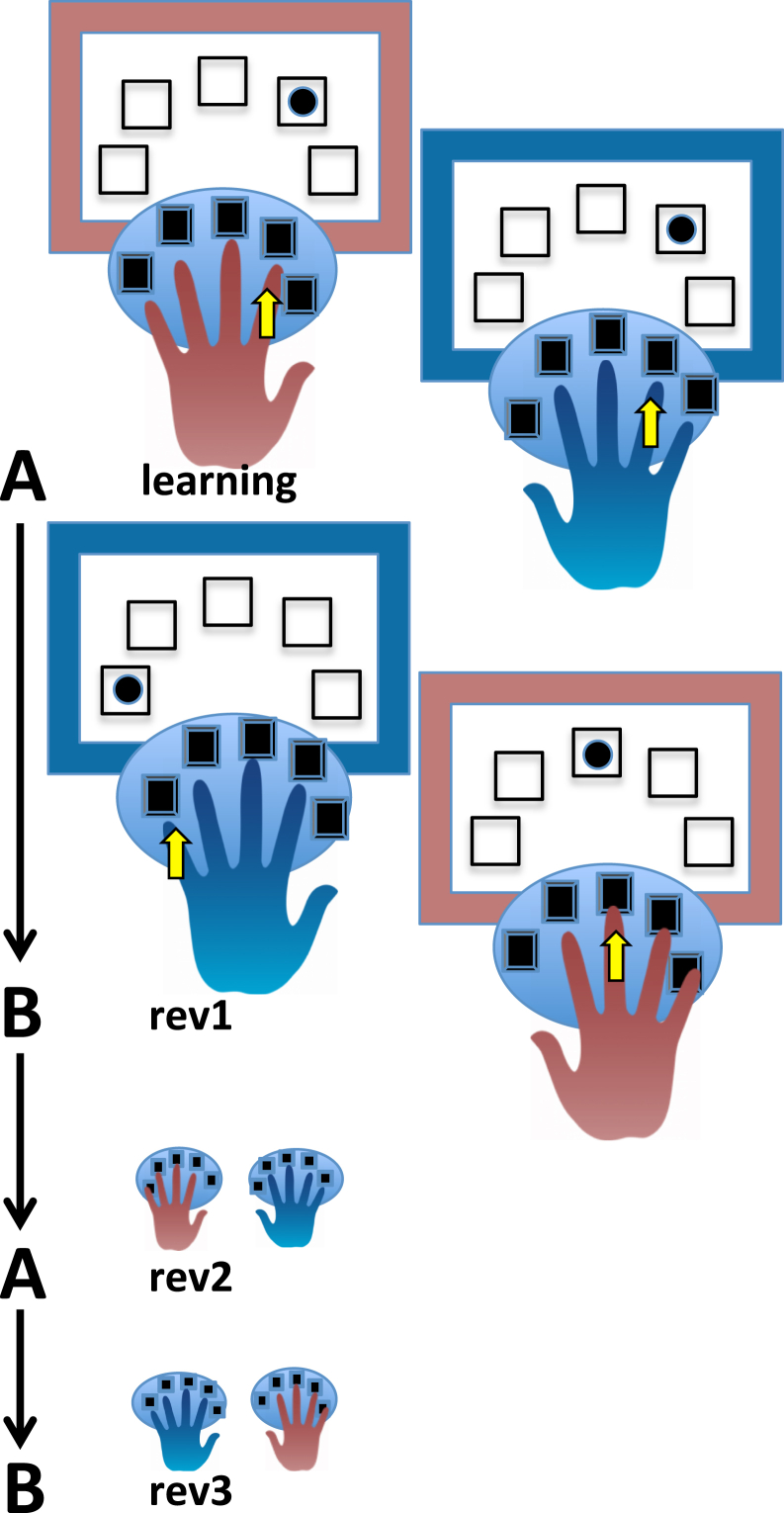
Novel deterministic reversal task with 3 hand reversals (rev). The color of the frame around the screen signals which hand to respond with, and the dot on the screen signals the correct finger **(A)**. At reversal, the frame-to-hand mapping changes; hence the color-hand rule is switched **(B)**. Thus, the originally learned condition **(A)** returns for the second reversal. Participants completed this task under 4 conditions: neutral (i.e., just informative)-punishment, neutral (informative)-reward, punishment-reward, and neutral (informative)-neutral (informative). See Figure S2 for an example where the color of the frame around the screen indicated whether a response had to be made with the left or right hand with 2 colors in each condition.

**Figure 2:**
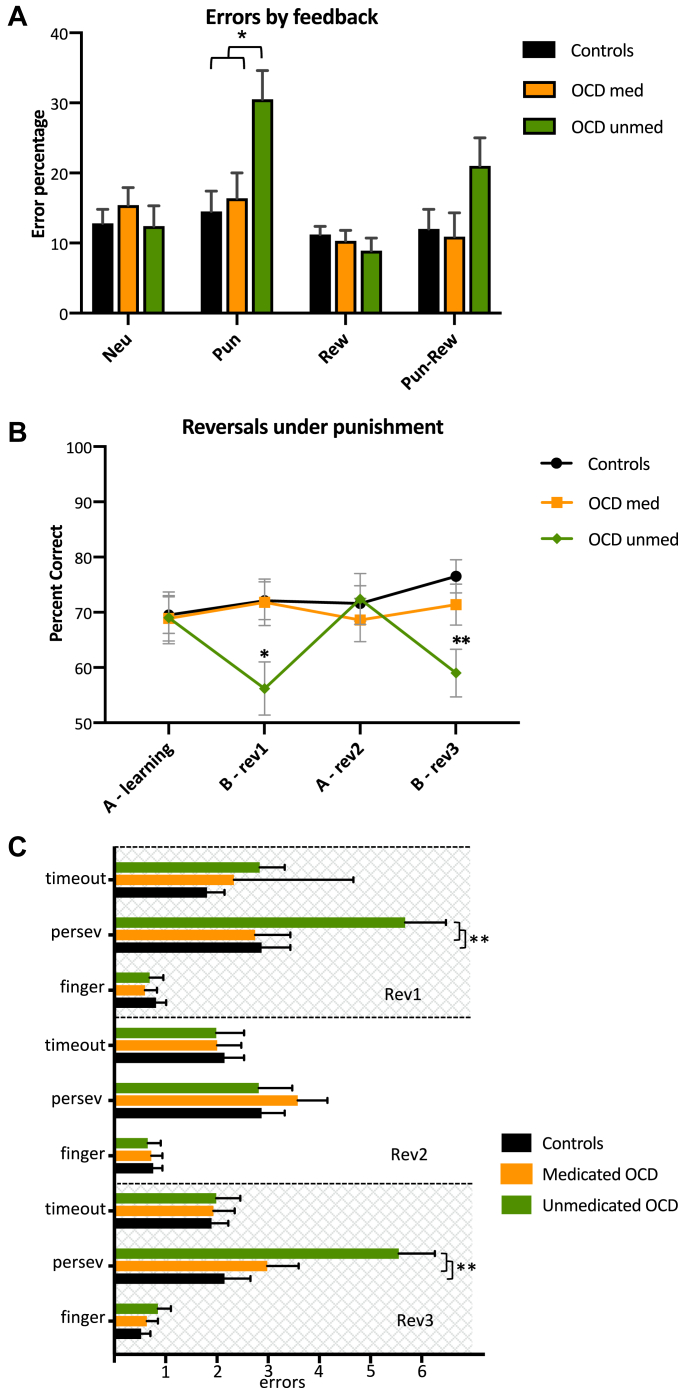
Deterministic reversal impairments in unmedicated (unmed) patients with obsessive-compulsive disorder (OCD). **(A)** Unmedicated patients with OCD were impaired with punishing feedback. Unmedicated patients with OCD made significantly more errors with punishing feedback than control participants and medicated (med) patients with OCD (*p* = .006 and *p* = .034, respectively). **(B)** Unmedicated patients with OCD were impaired only under the reversed rule. Unmedicated patients performed significantly worse than control participants (*p* = .024) on both reversals (revs) (*p* = .024, *p* = .004) and significantly poorer than medicated patients on the first reversal (*p* = .048) but not on the final reversal (*p* = .096). **(C)** Unmedicated patients with OCD were impaired due to perseverative errors (persev). Unmedicated patients with OCD made significantly more perseverative errors (responding with the original hand-color association) during both rev1 and rev3 compared with control participants and medicated patients, respectively (*p* = .014, *p* = .019; *p* = .001, *p* = .024). Finger denotes responding with the wrong finger, and timeout denotes failure to respond in time. ∗*p* < .05, ∗∗*p* < .01. Neu, neutral (just informative feedback); Pun, punishment; Rew, reward.

We developed a novel deterministic reversal learning task with a high level of difficulty (Figure 1). This task begins with an initial learning phase during which the participant learns to respond with either the right or left hand depending on the color of the screen’s frame and at the same time to respond to a target on the screen with the correct corresponding finger. Participants were initially trained to respond as quickly as they could on an instructed task version; monitoring their personal response speed places individuals under time pressure. Because the matching of digits is different for each hand (with the exception of the middle finger), additional executive load is created. Subsequently, there are 3 reversals (the second reversal uses the originally learned rule). This task was carried out using 3 types of feedback (2 being salient, using monetary punishment/reward combined with salient sounds, and 1 being neutral only, informing whether the response was correct/incorrect) (for further description, see Figure 2 and the Supplement).

### Probabilistic Reversal Learning Task

**Figure 3:**
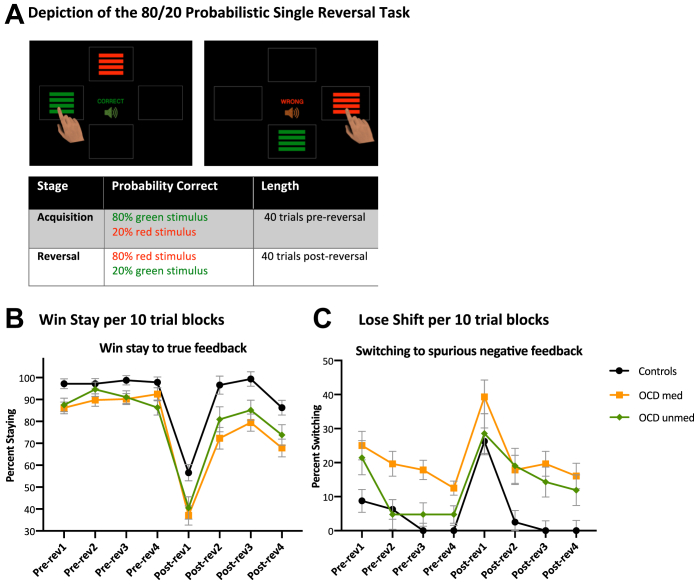
Probabilistic learning deficits in patients with obsessive-compulsive disorder (OCD). **(A)** Depiction of the touchscreen probabilistic reversal task. Stimulus A was correct on 80% of occasions and stimulus B on 20%, and this contingency reversed after 40 trials. The stimuli were counterbalanced and appeared at random in 1 of 4 locations on the screen. Each time the participant selected a stimulus, informative feedback was given (auditory and written) on the screen about whether the correct stimulus had been chosen. **(B)** Patients with OCD were impaired on win-stay behavior, not continuing with the 80% correct stimulus after positive feedback. Both medicated (med) and unmedicated (unmed) patients with OCD showed significantly less win-stay behavior than control participants before and after reversal (rev) (*p* = .005). **(C)** Medicated patients with OCD exhibited more lose-shift behavior before and after reversal, and unmedicated patients also exhibited more lose-shift behavior after reversal, shifting away from the 80% correct stimulus after receiving spurious (20% negative) feedback. Unmedicated patients performed similarly to control participants before reversal and switched less than medicated patients with OCD on prereversal blocks 2 and 3 (*p* < .05). However, after reversal, medicated and unmedicated patients both shifted significantly more than control participants during postreversal blocks 2, 3, and 4 (*p* < .05).

The probabilistic reversal task was self-paced. Participants were instructed to choose between 2 stimuli (red vs. green). During the initial 40 trials, one choice was usually (80%) correct, and the other was usually incorrect (20%) (80:20 acquisition phase); participants were instructed to expect that one stimulus would be correct more often. Feedback for correct trials was a high-pitched tone and a label stating “CORRECT,” while incorrect trials were followed by “INCORRECT” and a low-pitched tone. These contingencies were reversed for the subsequent 40 trials (Figure 3A).

The order of the 2 tasks was counterbalanced to control for possible transfer effects.

### Computational Modeling of Probabilistic Learning

To instantiate hypothesized cognitive processes generating the observed behavior and to facilitate quantitative comparisons, we fitted empirical behavioral data to a family of reinforcement learning models. These were value-based models featuring behaviorally interpretable parameters using various model-free approaches that have previously been shown to give parsimonious accounts of empirical behavior for the task [e.g., (12)]. We also included a simple model-based variant in which the subject took the antagonistic nature of the 2 stimuli that were available into account. The best model was found by bridge sampling model comparison, balancing fit and parameter parsimony (25). The winning model included distinct learning rates for rewarding and punishing outcomes, sensitivity to reinforcement-driven action values, and stimulus stickiness (repetition tendency) (for additional details, see the Supplement).

### Deterministic Reversal Analyses

In the deterministic reversal task, the learning block is followed by 3 reversals, and participants have to respond with the correct hand and finger under time pressure on each trial. We examined earnings related to performance (at the end of the task), accuracy (percentage of trials correct), reaction time (number of trials responded to within the time limit), occurrence of repeated errors (errors followed by an error), and the number of trials needed to reach the learning criterion (i.e., 4 correct answers in a row). These scores were analyzed by feedback (neutral informative, reward, or punishment) and by block (learning, reversal 1, reversal 2, reversal 3; each of 20 trials) using repeated measures analysis of variance (ANOVA) with group as a between-subjects variable. Post hoc pairwise analyses were performed (using the Šidák correction for multiple comparisons and the Huynh-Feldt method) to determine specific differences in performance between patients with OCD and healthy control participants.

## Results

### Deterministic Learning and Reversal in Medicated and Unmedicated Patients With OCD

#### Effects of Feedback on the Deterministic Learning and Reversal Task

Only unmedicated patients were impaired on the task, specifically due to errors made under the punishment condition (Figure 2A). Performance, measured as errors collapsed over the learning and 3 reversal stages, was analyzed using a mixed-design ANOVA with a within-subject factor of feedback (neutral, punishment, reward, mixed reward/punishment) and a between-subject factor of group (controls, medicated OCD, unmedicated OCD). There was a significant feedback × group interaction (*F*_4.4,195.75_ = 3.67, *p* = .005), as well as main effects of group (*F*_2,89_ = 3.16, *p* = .047) and feedback (*F*_2.2,195.75_ = 10.36, *p* < .001). Pairwise comparisons revealed that this interaction was driven by the punishment condition, under which the control participants and medicated patients with OCD made significantly fewer errors than unmedicated patients (*p* = .006 and *p* = .034, respectively) (Figure 2A). There were no significant differences in the other feedback conditions (neutral, reward, and combined reward/punishment [all *p*s > .05]).

#### Learning and Reversal Performance Under Punishment in Medicated and Unmedicated OCD

These results show that unmedicated patients were impaired under punishment when the color-hand association differed from the original learned association (Figure 2B). Accuracy under punishment was analyzed using a repeated measures ANOVA with a within-subject factor of stage (learning, reversal 1, reversal 2, reversal 3) and a between-subject factor of group. There was a significant stage × group interaction (with a significant cubic component) (*F*_5.26,233.85_ = 0.004, effect size *d* = 0.58). Pairwise comparisons revealed a significant effect on reversals 1 and 3 (i.e., those in which the contingency was reversed from the original), with unmedicated patients performing significantly worse than control participants on both reversals (*p* = .024, *p* = .004) and having a significantly lower accuracy than medicated patients on the first reversal (*p* = .048); this difference became nonsignificant on the final reversal (*p* = .096).

#### Error Types Under Punishment

These results show that impairments in unmedicated patients resulted from perseverative errors (Figure 2C). There are 3 type of errors in the deterministic reversal paradigm: 1) wrong hand (a perseverative error reflecting an incorrect/previous hand-color association), 2) wrong finger on the correct hand, and 3) time out (failure to respond promptly). The resulting multivariate ANOVA showed a significant group × error × reversal interaction, although only for perseverative errors reversal 1 (*F*_2,89_ = 4.99, *p* = .009) and reversal 3 (*F*_2,89_ = 7.66, *p* = .001). Pairwise comparisons demonstrated that unmedicated patients with OCD made significantly more perseverative errors than control participants and medicated patients during both reversal 1 (*p* = .014 and *p* = .019, respectively) and reversal 3 (*p* = .001 and *p* = .024, respectively).

#### Clinical Factors Related to Perseverative Responding

We tested the relationship between 3 key clinical scales for OCD (the Y-BOCS for obsessions/compulsions, the Montgomery–Åsberg Depression Rating Scale for depressive symptoms, and the Spielberger State-Trait Anxiety Inventory for anxiety symptoms) and perseverative reversal errors in medicated and unmedicated patients. Multiple regression was used to predict the percentage of perseverative (wrong hand) reversal errors from obsessive/compulsive symptoms, depressive symptoms, and anxiety symptoms. These variables were significant predictors of perseverative responding only in the medicated OCD group (*F*_3,27_ = 4.54, *p* = .012, *R*^2^ = 0.36). Y-BOCS scores added most significantly to the prediction (β = 0.62, *p* = .001).

### Probabilistic Reversal

#### Win-Stay Responding After Majority Correct Feedback

Both medicated and unmedicated patients with OCD exhibited significantly less win-stay behavior (i.e., repeating the response that was just rewarded) compared with control participants. Repeated measures ANOVA showed a significant group × reversal-stage interaction (*F*_2,86_ = 2.28, *p* = .005), reflecting a greater deficit in both patient groups (than control participants) after reversal. Pairwise tests confirmed that medicated patients with OCD showed significantly lower win-stay behavior (across all trials) than control participants, and unmedicated patients showed this deficit during all blocks except for the second block before reversal (Figure 3B).

#### Lose-Shift Responding After Minority Negative Feedback

Medicated patients with OCD exhibited significantly more lose-shift behavior (i.e., shifting to the alternative response immediately after nonreward) than control participants irrespective of reversal, while unmedicated patients with OCD also shifted significantly more than control participants after reversal. Repeated measures ANOVA showed a significant group by reversal block (10 trials each) interaction (*F*_2,86_ = 3.23, *p* = .044, effect size *d* = 0.38). Pairwise tests revealed significant increased shifting in medicated patients with OCD compared with control participants on blocks 2, 3, and 4 before reversal (*p* < .05) as well as in blocks 6, 7, and 8 after reversal (*p* < .05). Unmedicated patients performed similarly to control participants before reversal, shifting less than medicated patients with OCD on stages 2 and 3 (*p* < .05). However, after reversal, medicated and unmedicated patients shifted at a similar level, both significantly more than control participants, for stages 6, 7, and 8 (*p* < .05) (Figure 3C).

#### Probabilistic Reversal Computational Modeling Results

The best-performing model included distinct learning rates for positive and negative feedback plus parameters for reinforcement sensitivity (the overall impact on choice of reinforcement-driven action values) and stimulus stickiness.

Complementary-updating variants substantially outperformed their counterparts, as estimated by bridge sampling. A summary of the performances of all 6 computational models tested is provided in the Supplement, with the best-fitting model being {*α*_rew_,*α*_pun_,*τ*,*τ*_stim_}.

**Figure 4:**
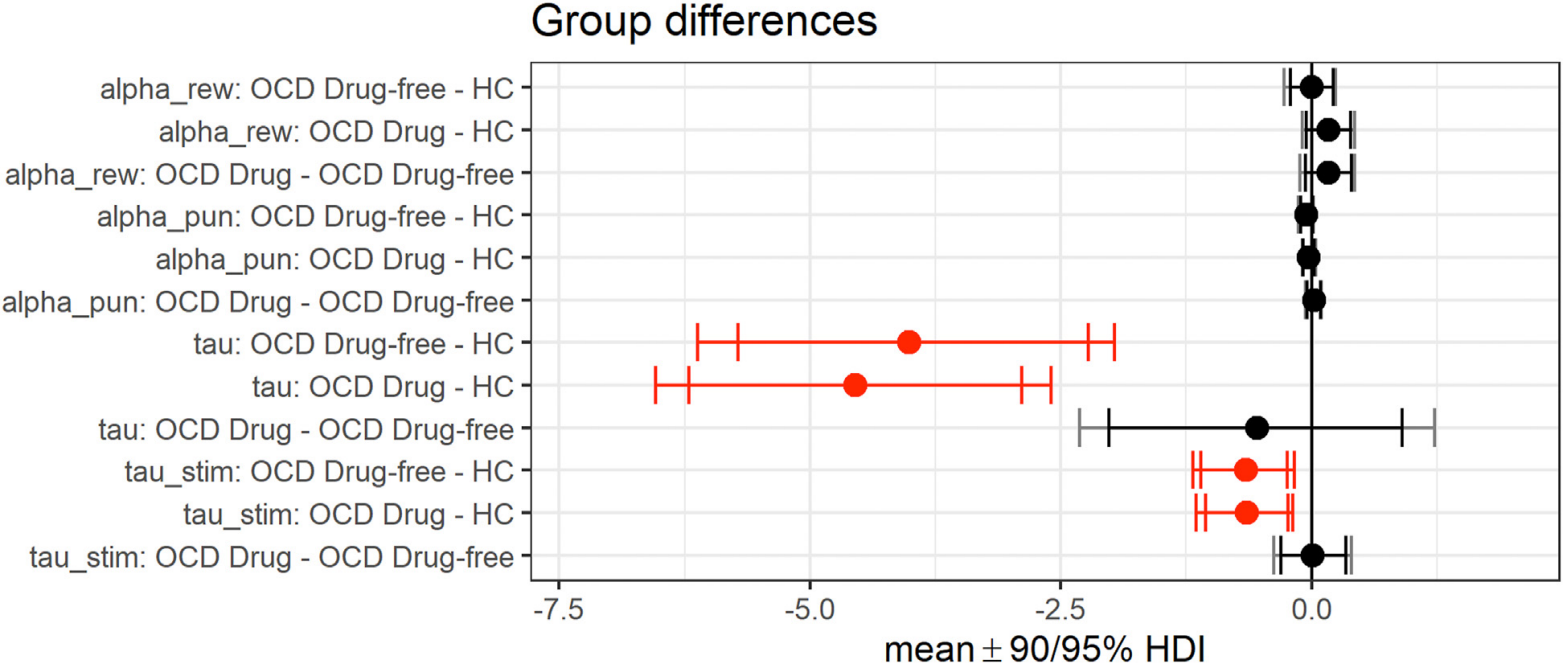
Group differences of the best-fit computational model of behavior. The parameters represent learning rate following reward outcomes (alpha_rew), learning rate following punishment outcomes (alpha_pun), reinforcement sensitivity (tau), and stimulus stickiness sensitivity (tau_stim). The updating rule instantiates a simple internal model of the 2-stimulus task, and choices were made according to a softmax choice rule. Error bars show the posterior distributions of group differences mean parameter values as highest posterior density intervals (HDIs). Red indicates that the 95% HDI (Bayesian credible interval) excludes 0. HC, healthy control; OCD, obsessive-compulsive disorder.

Both medicated and unmedicated patients with OCD had significant decreases in both reinforcement sensitivity and stimulus stickiness compared with healthy control participants. This signifies a more haphazard decision-making process and a higher tendency to switch from recently chosen stimuli (Figure 4).

Unlike for deterministic reversal learning, there was no relationship between either behavioral or computational measures of increased switching in patients with OCD and clinical measures (*p* > .05).

## Discussion

Unmedicated people with OCD had deficits in both deterministic and probabilistic reversal performance. For medicated patients with OCD, although there were no initial learning impairments or reversal deficits in deterministic rule learning, deficits were evident during probabilistic rule learning. A striking observation was that the deterministic reversal deficit in unmedicated patients with OCD was restricted to the initial and third reversals of the rule, but performance was intact on the second reversal. Given the return of the reversal deficit for the final stage in unmedicated patients with OCD, this indicates an inability to disengage from the initial learned rule. Moreover, we found that this perseverative tendency was correlated with symptom severity, suggesting that it underlies repetitive obsessions and compulsive behavior in OCD. The findings indicate that treatment with SSRIs may improve flexibility under certain conditions in reversal learning in patients with OCD, consistent with evidence concerning serotonin and cognitive flexibility (6,8,9).

Medicated and unmedicated patients with OCD showed equivalent deficits in response accuracy during probabilistic discrimination reversal, although only medicated patients were impaired during its initial learning. In both groups, the impairments were driven by a greater tendency to shift away from the mostly correct stimulus, especially after the spurious 20% negative feedback that occurred on a minority of the trials. These findings indicate that patients with OCD not only have a perseverative tendency, as indicated by the results on the deterministic task but also an apparently opposite tendency of behavioral shifting or switching under more stochastic reinforcement of probabilistic reversal. This tendency was also reflected in computational modeling showing that patients with OCD had reduced sensitivity to feedback and reduced stimulus stickiness. Thus, they exhibited suboptimal performance through a failure to maximize responding to the 80% rewarded stimulus, indicating that patients with OCD do not form an accurate representation of optimized responding based on probabilistic feedback (26). In general, the findings support the Bayesian model advanced by Fradkin *et al.* (14) that patients with OCD have special problems with state transitions, which may suggest that they have particular difficulties in detecting how sensations and events unfold in sequence, leading to problems of prediction and control. These problems are exacerbated under conditions of uncertainty, such as in the probabilistic reversal task, where there is a lack of absolute feedback clearly supporting the repetition of a specific policy—even though the amount of uncertainty is fixed and therefore can be anticipated (27). This enhanced uncertainty may promote exploratory tendencies for gathering further environmental feedback, as manifested by enhanced switching. By contrast, in deterministic reversal learning tasks, more perseverative habitual behavior is to be expected in familiar environments because of the availability of a well-learned routine or rule which has previously had consistent feedback. As Fradkin *et al.* stated, “This may explain why most habitual, repetitive compulsions occur in everyday situations (e.g. hand-washing, door-locking)” (14).

### Deterministic Reversal Learning

While deterministic reversal learning was impaired in unmedicated patients, the specific task used not only had a hierarchical nature in which response selection was governed by a conditional rule (red → left hand, green → right hand), but also a lower-order specific finger-location mapping. The deficit observed was related to the conditional rule rather than nonperseverative finger-mapping errors or failures to respond. These findings further emphasize the specificity of the impairment in OCD, which is related to cognitive rule inflexibility rather than to some more general aspect of performance monitoring. This is perhaps the first demonstration of cognitive inflexibility in patients with OCD in the context of relatively stable and clear environmental contingencies represented by a deterministic task. As well as being demanding, the task was performed under varying feedback conditions that included separate rewarding, punishing, and neutral conditions in view of previous literature suggesting that patients with OCD respond differentially to reward and punishment (12,13). While unmedicated patients with OCD did indeed exhibit the largest deficits in rule reversal under punishment, the absence of a deficit in initial learning suggests that the reversal impairment was not due to abnormal emotional reactions to punishment. Instead, punishment apparently led to enhanced learning of the initial rule, perhaps thereby interfering with subsequent reversal performance.

Remarkably, medicated patients were unimpaired in rule reversal, suggesting that SSRI medication remediated the way that punishment promoted inflexibility. These findings contrast with the previous, rather sparse, literature on the effects of SSRI medication on cognitive functioning in OCD (28, 29, 30), although a reinforcement learning study by Palminteri *et al.* (31) supported a beneficial role of SSRI medication in instrumental learning. The current findings are also consistent with an extensive animal literature showing specific perseverative impairments in reversal learning following local depletion of serotonin in the orbitofrontal cortex of marmoset monkeys (6,9,32) and rats (5,33,34) and remediation of reversal deficits following subchronic SSRI treatment (5,34).

### Probabilistic Reversal Learning

In contrast to deterministic reversal, SSRI medication was associated with impaired overall performance in OCD during the probabilistic reversal task. Detrimental shifting was generally increased in medicated patients, perhaps showing that increased flexibility conferred by chronic serotonergic medication, as shown in the deterministic reversal task, is not always beneficial. This conclusion is supported by the profound effects of acute administration of the SSRI escitalopram, which increased shifting in the same probabilistic reversal task in healthy volunteers (35). Bari *et al.* (36), using a similar rat model, also showed that acute low-dose citalopram increased shifting, but also that acute high-dose or subchronic citalopram had the opposite effect, reducing shifting and thereby improving probabilistic reversal learning. Consequently, one might have expected amelioration rather than exacerbation of detrimental shifting behavior when OCD was treated chronically with SSRIs. Indeed, it is not clear that medication was responsible for this detrimental shifting behavior because unmedicated patients with OCD also showed this propensity during reversal. Similarly, our computational modeling showed no differences in responding to probabilistic feedback in the medicated and unmedicated OCD groups, replicating findings from a multiple probabilistic reversal paradigm (12).

Like Remijnse *et al.* (37), we showed that patients with OCD had overall deficits in performance on the probabilistic reversal task, although that study did not report findings for the initial learning stage. Computational modeling of the probabilistic learning and reversal data showed that both OCD groups exhibited a general tendency toward reduced stimulus stickiness, indicating a greater propensity to switch responding on each trial regardless of feedback. The winning model also indicated a reduction in reinforcement sensitivity in both medicated and unmedicated OCD groups, which can also be interpreted as an enhanced tendency toward exploration versus exploitation (38). This could be viewed as the adoption of a response strategy that interferes with model-based learning of the reinforcement contingencies. The question remains why this strategy may be adopted. Anxiety is commonly evoked by uncertainty (39) and is a possible candidate to explain the OCD deficit. For example, it is plausible that patients with OCD lack confidence in their decisions in such volatile circumstances and adopt a maladaptive strategy of checking the outcomes associated with the alternative stimulus (16). However, it is one of the limitations of the current study that the sample size prevented us from performing structural equation modeling and mediation analysis to address this important question.

### Limitations

Evaluation of a treatment such as the SSRIs in this study is often best achieved using a within-subject crossover design, but we opted for a between-group design of medicated versus unmedicated patients instead because of likely confounding practice effects in tests of cognitive flexibility and the excessively lengthy period required for chronic SSRI medication and for its washout. Such a design requires careful matching of groups, and although the current Y-BOCS scores of the medicated group were matched at the time of testing, it is likely that these medicated patients had had more severe symptoms which had been ameliorated to some extent by SSRI medication. Nevertheless, the relative sparing of deterministic reversal in this group, compared with the unmedicated patients, was striking. The medicated patients with OCD were evidently more impaired in probabilistic learning than those in the unmedicated group, and this may also be related to their more severe underlying symptoms rather than to SSRI medication per se. However, it can be concluded that their deficit in probabilistic learning and reversal was not remediated by this serotonergic treatment.

### Conclusions

Using a novel deterministic reversal learning task, we demonstrated, for the first time, perseverative deficits in OCD that are remediated by SSRIs and related to severity of clinical symptoms. These data contrast with increased shifting, reduced overall sensitivity to feedback, and a reduced tendency to select previously chosen stimuli in a probabilistic reversal learning task in the same patients with OCD, whether medicated or unmedicated. We suggest that patients with OCD exhibit rigidity of rule-governed behavior following training under punishment in stable situations, which can be ameliorated by SSRIs, but they exhibit a treatment-resistant tendency to behavioral switching under conditions of feedback uncertainty in volatile environments, consistent with recent theoretical accounts. These findings have clinical significance in suggesting that SSRIs only remediate a subset of underlying cognitive impairments in OCD, and moreover that behavioral decision making in patients with OCD is likely to depend on the balance between familiarity and uncertainty in their environment and the nature of the reinforcing feedback for their choices that it provides.

## Supplementary Material

Key Resources Table
