# Supplementary material for: Perseveration and Shifting in Obsessive-Compulsive Disorder as a Function of Uncertainty, Punishment, and Serotonergic Medication
Source: Biol Psychiatry Glob Open Sci. 2023 Jul 13;4(1):326–35. doi: 10.1016/j.bpsgos.2023.06.004 (PMC10829647; doi:10.1016/j.bpsgos.2023.06.004)
Supplement: Supplementary Data [file mmc2.pdf]

SUPPLEMENTARY INFORMATION

Perseveration and Shifting in Obsessive–Compulsive Disorder as a Function of Uncertainty, Punishment, and Serotonergic Medication

Apergis-Schoute *et al.*

Contents

Supplementary Methods....2
Deterministic reversal learning task....2
Computational modeling of probabilistic reversal learning....5
Overview....5
Outline of computational methods....5
Models....6
Bayesian hierarchical modelling....8
Simulation and replication of model-independent behavioral measures....8
Parameter recovery from simulated data....9
Supplementary Results....9
OCD participants....9
Computational modeling of probabilistic reversal learning....10
The model that fitted the empirical data best was {α_{rew}, α_{pun}, τ, τ_{stim}}*....10
Reduced sensitivity parameters in patients with OCD....11
Simulation and replication of model-independent behavioral measures....11
Parameters recovered from simulated data generated by the winning model....13
Supplementary References....14

Supplementary Methods

Deterministic reversal learning task

Our novel deterministic reversal learning task was administered on a computer with the use of two hand boxes (see **Figure S1**).

Figure S1. *The five-finger hand boxes used for responding on the novel deterministic reversal learning task (designed by Apergis-Schoute and produced by the Biotronix workshop, University of Cambridge).*

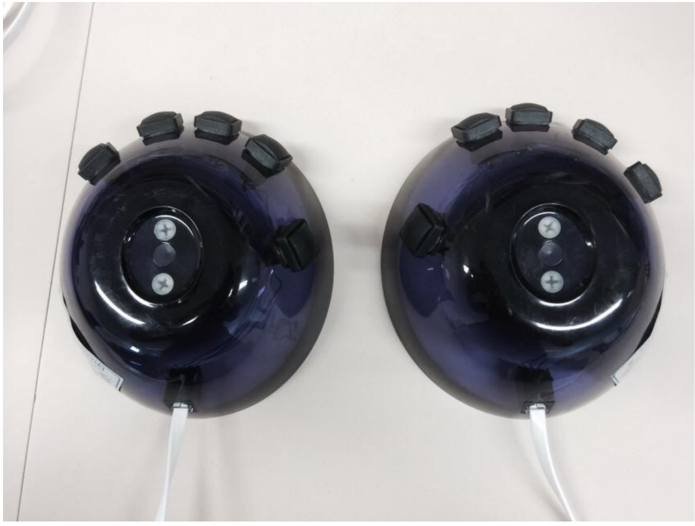

For each presentation, the computer screen showed five squares, which corresponded to five buttons on one of the hand boxes, with a dot in one of the squares to signal the correct response. The correct button had to be pressed within a certain time limit.

The color of the frame around the screen indicated whether a response had to be made with the left or right hand, with two colors used in each phase. Specimen trials are shown in **Figure S2**. This color–hand association reversed three times in each phase (giving four blocks in total per phase), thereby requiring flexible response behavior.

Figure S2. *Some possible screen presentations. Note: the word 'Left' or 'Right' was present for initial training but was not on the screen during the main experiment.* ***Trial 1:*** *red–left association; the left ring finger was the correct response, with a 50 pence reward and a positive sound delivered as the response was correct in this example.* ***Trial 2:*** *green–right association; the right index finger was the correct response, with 50 pence punishment and a negative sound delivered as the response was incorrect in this example.* ***Reversal trial 3:*** *red–right association; the right index finger was the correct response, with no reward as the response was too late in this example.* ***Trial 4:*** *green–left association; the left ring finger was the correct response.*

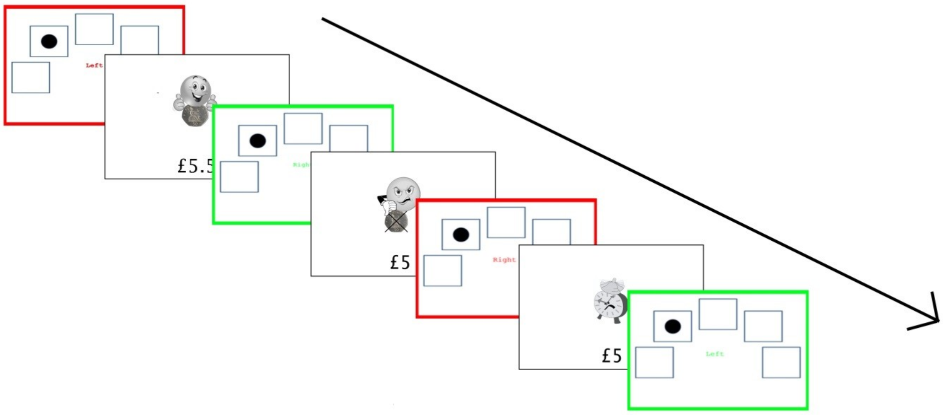

Across different phases of the experiment, the colors also signaled the trial type or feedback condition. There were three conditions possible for each trial: money to be earned (reward trials, with positive auditory feedback), potential monetary loss (punishment trials, with negative auditory feedback) or informative (no monetary gain or loss, with only visual feedback on performance). This design enabled us to investigate differences in flexible responding under rewarding, punishing and neutral (i.e. informative) conditions. Feedback conditions were paired in different ways across phases of the experiment (described below).

After a response (or response omission), the next screen showed the feedback, indicating whether the response was correct, incorrect, or too late, whether participants gained or lost money on that particular trial, and also the total amount of money the participant had earned up until then (see **Table S1**). After a correct response the subject was shown a positive emoticon of "thumbs up", and if it was a reward trial the subject was also presented with a 50-pence coin and a positive sound (and won 50 pence = £0.50). After an incorrect response the subject was shown a negative emoticon of "thumbs down" and if it was a punishment trial the subject was also presented with a crossed-out 50-pence coin and a negative sound (and lost 50 pence). If the subject was too late to respond the participant was shown an image of an angry clock, and if this was a punishment trial the participant also was shown a crossed-out 50-pence coin together with a negative sound to indicate monetary loss. The feedback screen was shown for 1500 ms, after which the next trial started.

Table S1. *Feedback for the different trial conditions.*

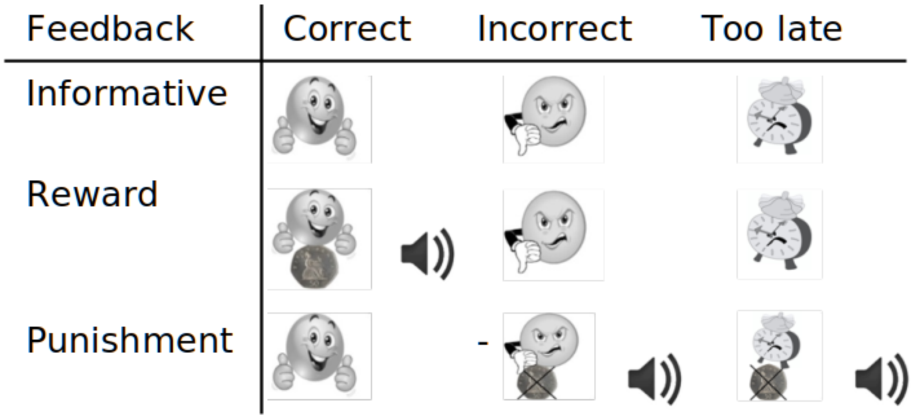

Prior to the actual experiment, the task started with three practice blocks of increasing difficulty. In the first practice block the words ‘Left’ or ‘Right’ were on the screen to help participants learn the correct hand association over 10 trials without a time limit.

From the second practice block onwards, the hand indication was no longer present, and the subjects now only had 1000 ms to respond. In order to continue to the next practice block, participants had to reach 80% accuracy (i.e., a maximum of 2 errors). The same cut-off of 80% accuracy was used in the third practice block, where participants had to respond correctly to at least 16 out of 20 trials. In this block, a personalized time limit was introduced, defined as 1.1 times the average reaction time from that participants’ second practice block, in order to put participants under pressure to respond as fast as possible. Following the third practice block, the same calculation was used to calculate the time limit for the actual experiment (based on this final practice block), which started after the criterion was reached.

This reversal learning experiment had four different phases, each with two different feedback conditions as described above (two of: informative, punishment, or reward) (see **Table S2**). The order of these phases was randomized. At the start of every phase, the color–feedback associations were made clear to the participants; however, the color–hand association had to be learned through trial and error. Color–feedback associations were consistent throughout the task and were counterbalanced between participants. In total, 8 separate colors were used. Each phase started with a block of 20 trials during which the correct color–hand association had to be learned. After the first block, the association was reversed without warning, so that participants then had to adjust their responding (see **Figure S2** for an example). The reversed association was used for the second 20-trial block, after which the color–hand association changed back to the original rule for the third block, and reversed again for the last (fourth) block. Each phase therefore consisted of four blocks of 20 trials each, producing 80 trials for each phase, giving 320 trials in total for each participant.

Table S2. *Organization of the different phases in our novel deterministic reversal learning task (presented in random order).*

Possible outcome	Combination of feedback conditions
Gain money	Reward–Informative
Loss of money	Punishment–Informative
Loss and gain of money	Punishment–Reward
No monetary feedback	Informative–Informative

Computational modeling of probabilistic reversal learning

Overview

We studied the effects of obsessive–compulsive disorder (OCD) and medication on performance in a two-block probabilistic reversal learning (PRL) task. In order to instantiate computational versions of the cognitive processes generating the observed behavior, and to facilitate quantitative comparisons, we fitted empirical behavioral data to a family of reinforcement learning models. These were value-based models featuring behaviorally interpretable parameters. We studied a number of "model-free" approaches which have previously been demonstrated to give parsimonious accounts of empirical behavioral data for the task. We also included a simple "model-based" variant in which the subject took into account the antagonistic nature of the two stimuli available. The best-fitting computational model was determined by bridge sampling comparison, balancing fit and parameter parsimony (detailed below). The best-performing model included distinct learning rates for rewarding and punishing outcomes, measures of reinforcement and stimulus "stickiness", and a simple internal model of the task described. Patients with OCD had significant decreases in both sensitivity to reinforcement and stimulus stickiness sensitivity, compared to healthy controls. This signified a higher tendency to switch from recently chosen stimuli and a more haphazard decision making process. There were, however, no significant differences between medicated and unmedicated OCD patients.

Outline of computational methods

In order to explain the observed selection behavior by participants, we instantiated several hypotheses describing their cognitive processes. We did this by fitting behavioral data to a set of reinforcement learning models whose parameters are interpretable in the context of behavior driving factors. This allowed for qualitative comparisons.

Concretely, our objectives were to

1. Find the best algorithmic description of the learning and decision-making process by bridge sampling model comparison.
2. For each group (namely healthy controls, unmedicated OCD patients and medicated OCD patients), estimate the parameter values that would most likely generate the empirical data, quantifying the driving factors for the winning model. We were especially interested in the posterior probability distributions of their group mean differences.
3. Verify that the winning model does indeed capture the empirical behavioral group differences and patterns. For each group, we simulated virtual subjects who performed the PRL task in silico and whose cognitive processes were instantiated by the winning model with the posterior group mean parameter values. We then retrodicted the model-independent, observable behavioral measures and analyzed them in the same way as we did for the empirical data (such as the win–stay/lose–shift analyses).
4. Check the interpretability of the models. Concretely, we fitted the winning model to simulated data and verified recovery of the generating parameters [1].

Models

To instantiate hypotheses on learning and decision making processes by the participants we focused on a family of models that learned by "model-free" reinforcement learning (essentially Q-learning [2] with the discount factor set to 0) and make choices according to the softmax choice rule, while allowing for tendencies to repeat recent choices. Kanen et al. [3] used these these models in their (human) study of reversal learning.

Clarke et al. [4] studied and compared this and other families of models in the context of reversal learning in marmosets, including:

- Random choice (no learning, for reference purposes).
- *n*-back analysis with a family of conditional logit regression models [5,6].
- Bayesian Bernoulli bandit using randomised probability matching [7], which approaches the exploration/exploitation trade-off by computing the posterior distribution of rewards and selecting an action according to the probability that it is optimal.
- Generalisation of the "model-free" learning model with reliability-modulated learning rates.
- Hybrid of the Bayesian Bernoulli bandit and model-free learning.

Amongst the set of models studied, "model-free" learning has been demonstrated to give the most parsimonious account of the empirical behavioral data, motivating our focus.

At their core, our models were composed of a learning process and a decision-making process (**Table S3**). For the learning process, our agent estimates a "value function" for the action of choosing a specific stimulus. After each trial, the chosen action's value is directly updated based on the discrepancy between the received and expected rewards. (Such methods, sometimes termed "model free", retrospectively estimate the values of actions based on the history of previous outcomes. This is in contrast to "model-based" algorithms, which prospectively predict the rewards of actions based on an internal "model" of the statistical structure of the environment.) The learned values inform the agent's choice on each trial, wherein the agent adopts a softmax choice rule [8,9] that balances exploration/exploitation.

Table S3: *Core model quantities, with learning and choice rules.*

Symbol	Definition
t	Trial number for a given subject.
α	Learning rate that determines the extent to which newly acquired information overrides old information; $\alpha \in [0, 1]$.
a_t	Action of choosing a given stimulus on trial t.
r_t	Reinforcement on trial t.
$Q^k{}_t$	Value of choosing stimulus k, such that only the value of choosing the chosen stimulus on trial t is updated by $Q^{\text{chosen}}{}_{t+1} = Q^{\text{chosen}}{}_t + \alpha(r_t - Q^{\text{chosen}}{}_t)$ initialised with $Q^k{}_0 \equiv 0$. Here, $r_t - Q^{\text{chosen}}{}_t$ is the prediction error.
τ	Reinforcement sensitivity, essentially the inverse temperature associated with stimulus value functions. It is restricted to ≥0 as the subjects' objective was to maximize their rewards by learning the stimulus values; $\tau \in [0, \infty)$.
$p^k{}_t$	Probability of choosing stimulus k on trial t, given by the softmax choice rule: $p_t^k = \frac{\exp(\tau Q_t^k)}{\sum_{i=1}^{K} \exp(\tau Q_t^i)}$

Building on this core model, we introduced various additional parameters (**Table S4**), instantiating distinct hypothetical cognitive processes at play.

Table S4: *Quantities in generalisations of the core model and their learning and choice rules.*

Symbol	Definition
α_{rew}, α_{pun}	In some models, α was split into distinct learning rates following reward and punishment outcomes, such that the α in the updating rule is replaced by α_{rew} or α_{pun} depending on the outcome (α_{rew} if $r>0$, α_{pun} if $r<0$).
$\mathbb{1}^k{}_t$	Indicator function of stimulus k being chosen on trial t (1 if chosen, 0 if not).
τ_{stim}	Stimulus stickiness sensitivity, essentially the inverse temperature associated with stimulus stickiness multiplied by a signed coefficient. In contrast to τ, τ_{stim} was allowed to take values <0, i.e. $\tau_{\text{stim}} \in (-\infty, \infty)$, signifying a tendency to “switch from” rather than “stick to” a previous choice. Including stimulus stickiness generalises the probability of choosing stimulus k on trial t to $$p_t^k = \frac{\exp\left(\tau Q_t^k + \tau_{\text{stim}} \mathbb{1}_{t-1}^k\right)}{\sum_{i=1}^K \exp\left(\tau Q_t^i + \tau_{\text{stim}} \mathbb{1}_{t-1}^i\right)}$$

The sensitivity parameters τ and τ_{stim} are related to the inverse temperature parameter $\beta \in [0, \infty)$ in the standard softmax choice rule, which controls the level of stochasticity in the agent’s choice, ranging from random choosing ($\beta = 0$) to pure exploitation ($\beta \to \infty$). In our case, in order to avoid parameter degeneracy, the inverse temperature was absorbed into the sensitivity parameters, such that τ and τ_{stim} take the form $\beta\times$(a signed coefficient). Note the latter factor may be <0. For example, in the case of stickiness, $\tau_{\text{stim}} < 0$ was permitted, to signify a tendency to switch rather than repeat.

We label models by their parameters. We initially tested a family of model-free reinforcement learning algorithms, adopted in existing reversal-learning literature [3]. These models were $\{\alpha, \tau\}$, $\{\alpha, \tau, \tau_{\text{stim}}\}$, $\{\alpha_{\text{rew}}, \alpha_{\text{pun}}, \tau\}$ and $\{\alpha_{\text{rew}}, \alpha_{\text{pun}}, \tau, \tau_{\text{stim}}\}$.

We then tested complementary-updating variants of the best-performing models, indicated by an asterisk: $\{\alpha_{\text{rew}}, \alpha_{\text{pun}}, \tau\}^*$ and $\{\alpha_{\text{rew}}, \alpha_{\text{pun}}, \tau, \tau_{\text{stim}}\}^*$. These models shared the same parameters as their counterparts, but their learning process was modified to follow a complementary update rule. On each trial, instead of updating only the value function of the chosen stimulus, the value function of the unchosen stimulus was also updated. (Values were initialised at 0.5.) This was to instantiate, simply, the subjects’ knowledge (from the verbal instructions given prior to their sessions) that one of the two stimuli is the optimal stimulus in a given block. Explicitly, the updating rule changes from

$$Q^{\text{chosen}}{}_{t+1} = Q^{\text{chosen}}{}_t + \alpha(r_t - Q^{\text{chosen}}{}_t) \qquad (1)$$

where only the value function of the chosen stimulus is updated and the value functions are initialised with $Q^k{}_0 \equiv 0$, for all k, to

$$Q^{\text{chosen}}{}_{t+1} = Q^{\text{chosen}}{}_t + \alpha(r_t - Q^{\text{chosen}}{}_t) \qquad (2)$$

$$Q^{\text{unchosen}}{}_{t+1} = 1 - Q^{\text{chosen}}{}_{t+1}$$

where the value functions are initialised with $Q^k{}_0 \equiv 0.5$, for all k.

(This complementary update rule was applicable to our specific task and implementation, in which subjects were informed beforehand that one of the two stimuli was optimal within a block of fixed contingencies, and abrupt reversal(s) of contingencies were to be anticipated. For more general reversal learning situations—such as tasks involving multiple stimuli, for example—this exact variant would not be applicable.)

Bayesian hierarchical modelling

Our study has an inherent hierarchical structure, with trial-level (trial-by-trial) data conceptually beneath a subject level (allowing for individual variations within a group) beneath a group level (group mean behavior). We addressed this property of our data using Bayesian hierarchical modelling [3,10]. Group level parameters were sampled from the following priors:

$$\alpha_{group}, \alpha_{group\_rew}, \alpha_{group\_pun} \sim \text{Beta}(1.2, 1.2) \qquad [11] \qquad (3)$$

$$\tau_{group} \sim \Gamma(4.82, 0.88) \qquad [12]$$

$$\tau_{stim\_group} \sim \text{N}(0, 1) \qquad [13]$$

Intersubject variability in the model parameters was based on the following priors:

$$\sigma_{\alpha}, \sigma_{\alpha rew}, \sigma_{\alpha pun}, \sigma_{\tau stim} \sim \text{half-N}(0, 0.05) \qquad (4)$$

$$\sigma_{\tau} \sim \text{half-N}(0, 1) \qquad [3,12]$$

where half-N is the normal distribution constrained to ≥ 0.

The trial-level behavior was then generated using subject-specific parameter values

$$\theta_{subj} \sim \text{N}(\theta_{group(subj)}, \sigma_{\theta})$$

where θ is the vector of model parameters. We implemented the learning and choice algorithms described above using these parameter values, and treated the two blocks in the reversal learning task (with opposite stimuli contingencies) as contiguous.

For each model, we estimated the parameter values that best fitted our empirical data, by approximating the (log-)posterior distribution over the parameters given the data and model using Hamiltonian Markov chain Monte Carlo sampling (HMCMC) implemented with Stan [14]. We checked that the potential scale reduction statistic $\hat{R}$ was close to 1, indicating that the 8 randomly initialized HMCMC sampling chains we used were well mixed, thus increasing our confidence in the parameter estimates [1,15,16].

We used bridge sampling [17,18] to compare our models to discern which amongst those tested was most likely (and parsimoniously) to have generated the empirical data, assuming equal prior model probabilities. Bridge sampling allowed us to directly estimate the Bayesian evidence (marginal likelihood of the data given a model) and the marginalisation over parameter space meant that free parameters were automatically penalized. In other words, unnecessary model complexity was automatically penalized, guarding against overfitting that could lead to poor generalisation, and acting as an automatic Occam's razor [18–20].

Simulation and replication of model-independent behavioral measures

To verify that the hypothesis instantiated by the winning model $\{\alpha_{rew}, \alpha_{pun}, \tau, \tau_{stim}\}^*$ could capture the underlying cognitive processes generating the empirical behavior, we used the winning model to simulate the behavior of subjects in the experiment. For each group, we simulated 100 subjects using their previously fitted group-specific model parameter values. Model-independent behavioral measures derived from the simulated data were analyzed the same way as those from empirical data. This allowed for testable predictions and comparison of the behavioral patterns derived from the empirical and model-generated data.

To visualize the simulated results, we looked at four model-independent measures: the probability of repeating a choice following a positive feedback (win–stay), P(stay|win); the probability of switching to the other stimulus following a negative feedback (lose–shift), P(shift|lose); the probability of choosing the sub-optimal stimulus within the given block, P(error); and the number of perseverance errors—the number of consecutive trials, starting from the second post-reversal trial (the second trial in block 2), in which the optimal stimulus from the pre-reversal block (block 1) was chosen.

These measures were analyzed in the same way we analyzed our empirical data. In particular, we plotted the group mean of each behavioral measure and its standard error (SEM) for each block (before and after reversal) in a session. We also performed pairwise null hypothesis tests on the sessional group mean differences, accounting for multiple comparisons using Tukey's honestly significant difference procedure [21,22]. The significance level was set to 5% and the tests we implemented using the *anova1* and *multcompare* functions in the MATLAB Statistics and Machine Learning Toolbox [23].

Parameter recovery from simulated data

We verified that the fitted parameter values were meaningful, i.e. that they were not degenerate. We fitted the winning model to our simulated data, using the same priors. (The simulated data was generated by the winning model with the group-specific parameter values previously fitted to empirical data, without intersubject variability.) We then checked whether we were able to 'recover' the previously fitted parameter values that generated the data, by considering the mean and the 95% highest posterior density intervals (HDI) of their posterior distributions. Failure to recover the generative parameters could suggest that the experiment was underpowered to assess our model or that there was parameter degeneracy, for instance if model parameters were trading off against one another.

Supplementary Results

OCD participants

Medicated OCD participants were mostly being treated with selective serotonin reuptake inhibitors (SSRIs); see **Table S5** for medication details per patient.

Table S5: *Details of medications for patients with OCD (n = 28). Each line indicates one patient; total daily doses shown.*

sertraline 150 mg
paroxetine 60 mg
sertaline 150 mg
fluoxetine 20 mg
citalopram 16 mg
fluvoxamine 300 mg
sertraline 50 mg
fluvoxamine 300 mg
citalopram [dose unknown] + mirtazapine 45 mg
sertraline 200 mg
paroxetine 60 mg + mirtazapine 30 mg + clotiapine 50 mg
sertraline 50 mg
sertraline 200 mg
tramadol 100 mg + gabapentin 660 mg
sertraline 200 mg
fluoxetine 60 mg
escitalopram 20 mg
sertraline 50 mg
clomipramine 75 mg
sertraline 250 mg + risperidone 2 mg
sertraline 150 mg
fluvoxamine 300 mg + clotiapine 150 mg
citalopram 20 mg
sertraline 50 mg
sertraline 100 mg
sertraline 200 mg
sertraline 200 mg
sertraline 200 mg

Computational modeling of probabilistic reversal learning

The model that fitted the empirical data best was $\{\alpha_{rew}, \alpha_{pun}, \tau, \tau_{stim}\}$*

The complementary-updating variants vastly outperformed their counterparts, by approximately 30 orders of magnitude in posterior probabilities as estimated by bridge sampling. A summary of the performances of all six computational models tested are listed in **Table S6**, with the best-fitting model being $\{\alpha_{rew}, \alpha_{pun}, \tau, \tau_{stim}\}$*.

Table S6: *Bridge sampling model comparison results. The winning model was {α_{rew}, α_{pun}, τ, τ_{stim}}*.*

	$\{\alpha, \tau\}$	$\{\alpha, \tau, \tau_{\text{stim}}\}$	$\{\alpha_{\text{rew}}, \alpha_{\text{pun}}, \tau\}$	$\{\alpha_{\text{rew}}, \alpha_{\text{pun}}, \tau, \tau_{\text{stim}}\}$	$\{\alpha_{\text{rew}}, \alpha_{\text{pun}}, \tau\}^*$	$\{\alpha_{\text{rew}}, \alpha_{\text{pun}}, \tau, \tau_{\text{stim}}\}^*$
Rank	5	6	3	4	2	1
Log marginal likelihood	-1274.2	-1276.1	-1267.9	-1269.5	-1203.9	-1199.7
Log posterior $\mathbb{P}(\text{model})$	-74.5	-76.5	-68.2	-69.8	-4.2	0.0
Posterior $\mathbb{P}(\text{model})$	4.2×10^{-33}	6.2×10^{-34}	2.4×10^{-30}	4.9×10^{-31}	0.015	1.0
Maximum $\hat{R}$	1.02	1.02	1.00	1.00	1.01	1.00

Reduced sensitivity parameters in patients with OCD

OCD patients had significantly reduced reinforcement sensitivity and stimulus stickiness sensitivity, compared to healthy controls (**Figure S3**). The reduced stimulus stickiness sensitivity in patients was also found in a previous study [3] and signified a higher tendency in OCD patients to switch away from recently chosen stimuli, regardless of feedback. Both sensitivity parameters pertain to the decision-making process and their observed reduction in magnitude demonstrated that patients employed a more haphazard decision-making process compared to healthy controls. No significant drug effects were observed, however.

Figure S3 *(duplicating **Figure 4** in the main article): Group mean differences for the model {α_{rew}, α_{pun}, τ, τ_{stim}}*. Error bars show the posterior distributions of group differences in group mean parameter values, as highest density intervals (HDI). Red indicates that the 95% HDI excludes 0.*

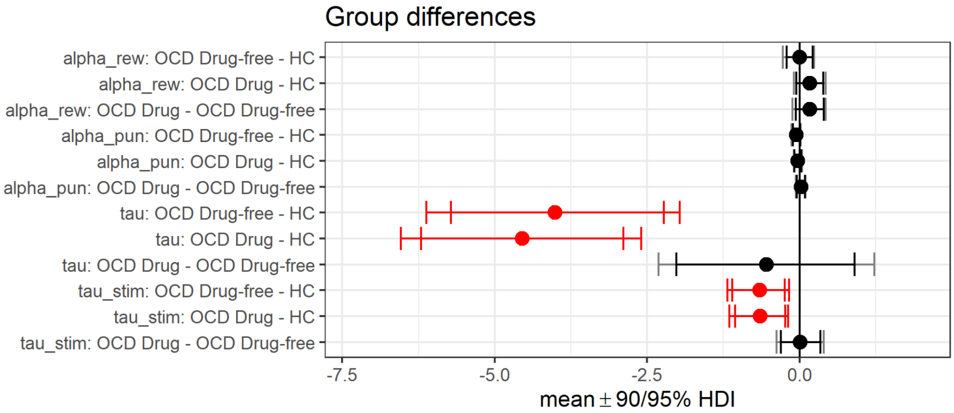

Simulation and replication of model-independent behavioral measures

The group means of each behavioral measure in each block of the experiment are presented in **Figure S4**. The significant group differences over a session (both blocks) are presented in **Table S7 and Table S8**. It is worth noting that the Tukey method is most conservative when the different groups have unequal sample sizes [24], which is true for our empirical data. The empirical group sample sizes were also considerably smaller than their simulated counterparts.

Figure S4: *Model-independent analyses of empirical behavioral measures and simulated measures generated by model {α_{rew}, α_{pun}, τ, τ_{stim}}* to check if the winning model accounted for the qualitative patterns in the empirical data. Points show group means and error bars are ±SEM.*

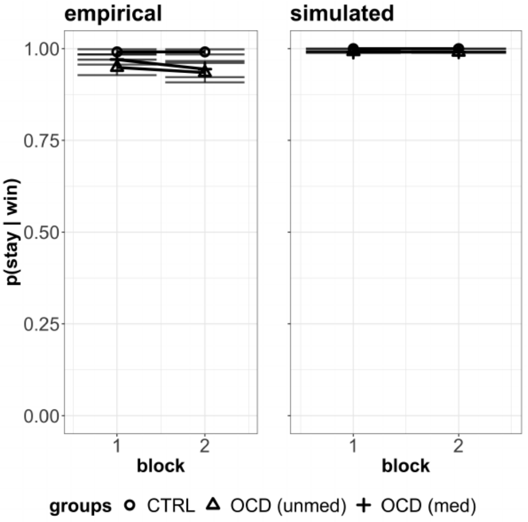

(a) $\mathbb{P}(\text{stay}|\text{win})$

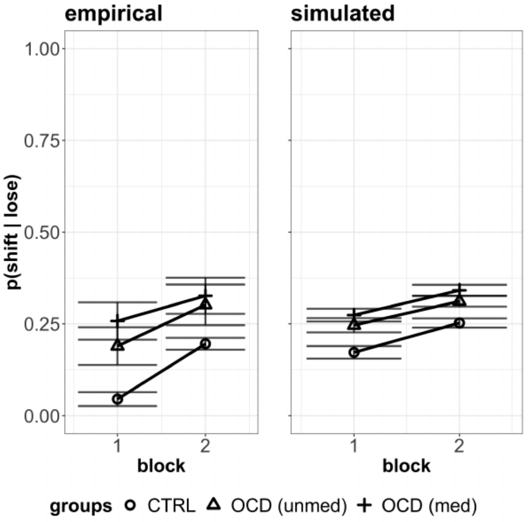

(b) $\mathbb{P}(\text{shift}|\text{lose})$

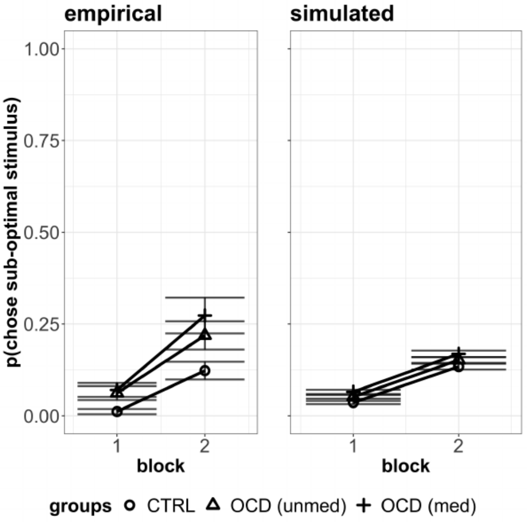

(c) $\mathbb{P}(\text{error})$

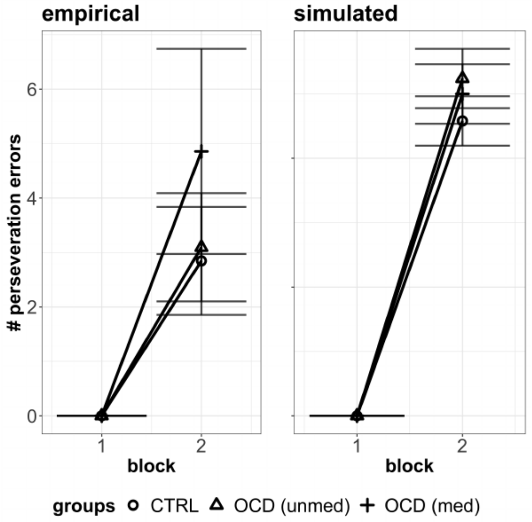

(d) No. perseverance errors

Behavioral replication by frequency (**Table S7**) was decent but not exact, the one deviation being the group difference in lose–shift behavior between healthy controls and unmedicated OCD patients. Simulated data indicated a significantly higher lose–shift count in patients, whereas while the empirical data suggested the same trend (group mean difference > 0), the difference was not significant.

Table S7: *(FREQUENCY) Significant ($p < 0.05$) group differences in model-independent behavioral measures for empirical data and data simulated from the winning model {α_{rew}, α_{pun}, τ, τ_{stim}}*. "+" and "−" are the signs of the corresponding significant group mean difference; "o" indicates no significant group mean difference ($p \geq 0.05$). Amongst the models tested, this one reproduced the empirical behavioral measure patterns best.*

Measure	Group difference	Empirical	Simulated $\{\alpha_{\text{rew}}, \alpha_{\text{pun}}, \tau, \tau_{\text{stim}}\}^*$
# Win−stay events	OCD (unmed) − CTRL	o	o
	OCD (med) − CTRL	−	−
	OCD (med) − OCD (unmed)	o	o
# Lose−shift events	OCD (unmed) − CTRL	o	+
	OCD (med) − CTRL	+	+
	OCD (med) − OCD (unmed)	o	o
# Total errors	OCD (unmed) − CTRL	o	o
	OCD (med) − CTRL	+	+
	OCD (med) − OCD (unmed)	o	o
# Perseverance errors	OCD (unmed) − CTRL	o	o
	OCD (med) − CTRL	o	o
	OCD (med) − OCD (unmed)	o	o

Behavioral replication by probability (**Table S8**) was not exact, with deviations where the simulated data suggested significant group differences absent in the empirical data. One deviation pertained to the group difference between healthy controls and unmedicated OCD patients in the probability of choosing the suboptimal stimulus. The others pertained to the group differences between healthy controls and both groups of OCD patients in the probability of win–stay. For all deviations, the empirical data showed consistent trends as the simulated data (group mean differences with the same signs) but were not significant.

Table S8: *(PROBABILITIES) Significant ($p < 0.05$) differences in model-independent behavioral measures for empirical data and data simulated from the winning model. Conventions as for **Table S7.** Amongst the models tested, this one reproduced the empirical patterns best.*

Measure	Group difference	Empirical	Simulated $\{\alpha_{\text{rew}}, \alpha_{\text{pun}}, \tau, \tau_{\text{stim}}\}^*$
$\mathbb{P}(\text{stay}\|\text{win})$	OCD (unmed) − CTRL	o	−
	OCD (med) − CTRL	o	−
	OCD (med) − OCD (unmed)	o	o
$\mathbb{P}(\text{shift}\|\text{lose})$	OCD (unmed) − CTRL	+	+
	OCD (med) − CTRL	+	+
	OCD (med) − OCD (unmed)	o	o
$\mathbb{P}(\text{error})$	OCD (unmed) − CTRL	o	+
	OCD (med) − CTRL	+	+
	OCD (med) − OCD (unmed)	o	o

Parameters recovered from simulated data generated by the winning model

Previously fitted parameters used to generate the simulated data, and their corresponding recovered values, are presented in **Table S9**. All generative parameter values fell strictly within their corresponding recovered 95% highest posterior density intervals (HDI) and all 8 randomly initialised sampling chains were well mixed, as indicated by the convergence diagnostic $\hat{R} \approx 1$ for every model parameter.

Table S9: *Parameter recovery analysis, using simulated data generated by model {α_{rew}, α_{pun}, τ, τ_{stim}}* with parameter values that best fitted our empirical data as estimated by Hamiltonian MCMC. The same model was then fitted to this simulated data to check that we could recover the simulated parameter values in this ideal (no intersubject variability) case. Here the recovered parameters are presented as "μ[a, b] ($\hat{R}$)" where μ is the posterior mean, [a, b] is the 95% HDI, and $\hat{R}$ is the potential scale factor reduction measure of convergence. Each simulated parameter fell within their corresponding recovered 95% HDIs and all 8 randomly initialised sampling chains were well mixed.*

Group	Parameter	Empirical best-fit	Parameter recovery $\mu[a, b]$ $(\hat{R})$
CTRL	α_{rew}	0.629	0.693 [0.622, 0.768] (1.001)
	α_{pun}	0.313	0.313 [0.299, 0.327] (1.001)
	τ	9.259	9.833 [8.940, 10.795] (1.002)
	τ_{stim}	0.847	0.851 [0.559, 1.152] (1.002)
OCD (unmed)	α_{rew}	0.630	0.592 [0.537, 0.653] (1.000)
	α_{pun}	0.262	0.256 [0.240, 0.273] (1.001)
	τ	5.249	5.359 [5.018, 5.708] (1.001)
	τ_{stim}	0.194	0.119 [-0.102, 0.340] (1.001)
OCD (med)	α_{rew}	0.800	0.788 [0.717, 0.863] (1.002)
	α_{pun}	0.285	0.290 [0.271, 0.310] (1.000)
	τ	4.709	4.583 [4.310, 4.861] (1.002)
	τ_{stim}	0.205	0.269 [0.074, 0.458] (1.001)

Supplementary References

1. Wilson RC, Collins AG. Ten simple rules for the computational modeling of behavioral data. eLife. 2019 26;8:e49547.

2. Watkins CJCH, Dayan P. Q-learning. Mach Learn. 1992 May 1;8(3):279–92.

3. Kanen JW, Ersche KD, Fineberg NA, Robbins TW, Cardinal RN. Computational modelling reveals contrasting effects on reinforcement learning and cognitive flexibility in stimulant use disorder and obsessive-compulsive disorder: remediating effects of dopaminergic D2/3 receptor agents. Psychopharmacology (Berl). 2019 Jul 20;236(8):2337–58.

4. Clarke HF, Cardinal RN, Rygula R, Hong YT, Fryer TD, Sawiak SJ, et al. Orbitofrontal dopamine depletion upregulates caudate dopamine and alters behavior via changes in reinforcement sensitivity. J Neurosci Off J Soc Neurosci. 2014 May 28;34(22):7663–76.

5. Lau B, Glimcher PW. Dynamic response-by-response models of matching behavior in rhesus monkeys. J Exp Anal Behav. 2005 Nov;84(3):555–79.

6. Seymour B, Daw ND, Roiser JP, Dayan P, Dolan R. Serotonin selectively modulates reward value in human decision-making. J Neurosci Off J Soc Neurosci. 2012 Apr 25;32(17):5833–42.

7. Scott SL. A modern Bayesian look at the multi-armed bandit. Appl Stoch Models Bus Ind. 2010;26:639–58.

8. Bridle JS. Training stochastic model recognition algorithms as networks can lead to maximum mutual information estimation of parameters. In: Proceedings of the 2nd International Conference on Neural Information Processing Systems. Cambridge, MA, USA: MIT Press; 1989. p. 211–7. (NIPS'89).

9. Boltzmann L. Studien über das Gleichgewicht der lebendigen Kraft zwischen bewegten materiellen Punkten. 1868. 1 p.

10. Daw ND. Trial-by-trial data analysis using computational models. In: Delgado MR, Phelps EA, Robbins TW, editors. Oxford: Oxford University Press; 2011. (Attention and Performance XXIII).

11. den Ouden HEM, Daw ND, Fernandez G, Elshout JA, Rijpkema M, Hoogman M, et al. Dissociable effects of dopamine and serotonin on reversal learning. Neuron. 2013 Nov 20;80(4):1090–100.

12. Gershman SJ. Empirical priors for reinforcement learning models. J Math Psychol. 2016;71:1–6.

13. Christakou A, Gershman SJ, Niv Y, Simmons A, Brammer M, Rubia K. Neural and psychological maturation of decision-making in adolescence and young adulthood. J Cogn Neurosci. 2013 Nov;25(11):1807–23.

14. Carpenter B, Gelman A, Hoffman MD, Lee D, Goodrich B, Betancourt M, et al. Stan: A Probabilistic Programming Language. J Stat Softw. 2017;76:1–32.

15. Gelman A, Carlin JB, Stern HS, Dunson DB, Vehtari A, Rubin DB. Bayesian Data Analysis. Third. Boca Raton, FL: CRC Press; 2013.

16. Brooks SP, Gelman A. General Methods for Monitoring Convergence of Iterative Simulations. J Comput Graph Stat. 1998 Dec 1;7(4):434–55.

17. Gronau QF, Singmann H, Wagenmakers EJ. bridgesampling: An R Package for Estimating Normalizing Constants. J Stat Softw. 2020 Feb 27;92:1–29.

18. Gronau QF, Sarafoglou A, Matzke D, Ly A, Boehm U, Marsman M, et al. A tutorial on bridge sampling. J Math Psychol. 2017 Dec 1;81(Supplement C):80–97.

19. Kass RE, Raftery AE. Bayes Factors. J Am Stat Assoc. 1995 Jun 1;90(430):773–95.

20. Pooley CM, Marion G. Bayesian model evidence as a practical alternative to deviance information criterion. R Soc Open Sci. 2018 Mar;5(3):171519.

21. Tukey JW. The problem of multiple comparisons. Princeton University; 1953.

22. Benjamini Y, Braun H. John W. Tukey's Contributions to Multiple Comparisons. Ann Stat. 2002;30(6):1576–94.

23. The MathWorks, Inc. MATLAB Statistics and Machine Learning Toolbox: User's Guide (r2019b) [Internet]. Natick, MA, USA; 2019. Available from: https://uk.mathworks.com/products/statistics.html

24. Somerville PN. On the conservatism of the Tukey-Kramer multiple comparison procedure. Stat Probab Lett. 1993 Apr 8;16(5):343–5.
